# TRIM25-mediated ferroptosis resistance is closely associated with poor prognosis in hepatocellular carcinoma

**DOI:** 10.3389/fimmu.2026.1792807

**Published:** 2026-04-01

**Authors:** Yulang Jiang, Peizhen Ma, Xuling Liu, Dong Li, Lili Xu, Ningning Liu, Mingyu Sun

**Affiliations:** 1 Shuguang Hospital Affiliated to Shanghai University of Traditional Chinese Medicine, Shanghai, China; 2 Shanghai University of Traditional Chinese Medicine, Shanghai, China; 3 Key Laboratory of Liver and Kidney Diseases, Institute of Liver Diseases, Shuguang Hospital Affiliated to Shanghai University of Traditional Chinese Medicine, Shanghai, China; 4 Department of Oncology, Shuguang Hospital Affiliated to Shanghai University of Traditional Chinese Medicine, Shanghai, China; 5 Department of Infectious Disease, Putuo Hospital, Shanghai University of Traditional Chinese Medicine, Shanghai, China

**Keywords:** bioinformatics analysis, ferroptosis, hepatocellular carcinoma, prognosis, TRIM25

## Abstract

**Objective:**

TRIM25 has been reported to promote hepatocellular carcinoma (HCC) cell survival by activating the Keap1-Nrf2 pathway. However, its expression profile in clinical HCC specimens and its potential role in regulating ferroptosis remain to be elucidated. This study aimed to determine the expression pattern of TRIM25 in primary HCC and its association with clinicopathological features and prognosis, utilizing both bioinformatic analysis and experimental validation in clinical samples and cell lines. In parallel, we investigated the regulatory effect of TRIM25 on ferroptosis in HCC cells, thereby offering experimental insights that could inform prognosis assessment and the development of targeted therapies for HCC.

**Methods:**

To investigate the expression pattern of TRIM25 in hepatocellular carcinoma and its clinical relevance, we obtained RNA-seq data and corresponding clinical staging information from The Cancer Genome Atlas. After data normalization, the Kruskal–Wallis H test was applied to assess TRIM25 expression differences across tumor stages. Transcriptomic data were also retrieved from the International Cancer Genome Consortium, and following normalization, the Wilcoxon rank-sum test was used to compare TRIM25 expression between HCC tumors and matched adjacent non-tumor tissues. Overall survival was evaluated in the TCGA cohort using Kaplan–Meier curves, with comparisons between high- and low-TRIM25 expression groups conducted via the log-rank test. Paired tumor and adjacent tissues from 12 HCC patients who underwent curative resection were collected. TRIM25 expression was analyzed at the protein level by Western blot and immunohistochemistry, and at the mRNA level by RT-qPCR. *In vitro*, liver cancer cell models with TRIM25 knockdown or overexpression were established. The effects of TRIM25 modulation on cell proliferation and ferroptosis susceptibility were assessed using CCK-8 assays, colony formation assays, and ferroptosis-related biochemical markers.

**Results:**

In the TCGA cohort, TRIM25 expression was significantly higher in HCC tissues than in adjacent normal tissues and progressively increased with advancing tumor stage. This finding was further validated in the ICGC cohort, in which TRIM25 expression was also significantly higher in tumor tissues than in matched adjacent non-tumor tissues. In the TCGA cohort, high TRIM25 expression was significantly associated with shorter overall survival in patients with HCC. Clinical specimen analysis confirmed that TRIM25 was upregulated in HCC tissues at both mRNA and protein levels. *In vitro*, TRIM25 silencing suppressed liver cancer cell proliferation and increased ferroptosis-related phenotypes, as indicated by increased ferrous iron and MDA levels, reduced GSH content and SOD activity, and elevated ROS and lipid peroxidation. Conversely, TRIM25 overexpression promoted proliferation, attenuated ferroptosis-related phenotypes, and reduced oxidative stress.

**Conclusion:**

TRIM25 is highly upregulated in HCC and significantly associated with poor clinical prognosis. Functionally, TRIM25 promotes tumor cell survival by reducing the sensitivity of HCC cells to ferroptosis. These findings suggest that TRIM25 may serve as a promising prognostic indicator and a potential therapeutic target for modulating ferroptosis pathways in HCC.

## 1 Introduction

Primary HCC is currently the sixth most diagnosed malignancy and the third leading cause of cancer-related death worldwide, with hepatocellular carcinoma accounting for the vast majority of cases and imposing a particularly heavy burden in Asia (2, 3). The major risk factors include chronic infection with hepatitis B or C virus, non-alcoholic fatty liver disease, long-term alcohol consumption, and exposure to aflatoxins. Recent epidemiological studies indicate that, with the continuous accumulation of patients with metabolic liver disease and cirrhosis secondary to viral hepatitis, the global incidence and mortality rates of hepatocellular carcinoma are projected to keep rising (4). Although comprehensive treatment strategies—such as surgical resection, local ablation, liver transplantation, molecular targeted agents, and immune checkpoint inhibitors—have been developed, data from the latest NCCN guidelines show that the 5-year survival rate for stage III hepatocellular carcinoma remains only around 30% to 60%, drops to less than 20% among patients with intermediate to advanced HCC in China; the prognosis of stage IV HCC is even worse. Moreover, even among early-stage patients who undergo curative resection, the postoperative recurrence rate within 1–3 years remains as high as 30%–50%. Drug resistance and tumor recurrence thus remain the major bottlenecks limiting the efficacy of HCC treatment, highlighting an urgent need to explore novel cell death pathways and molecular targets for effective therapeutic intervention (5).

Back in 2012, Dixon et al. initially proposed ferroptosis, defining it as a newly identified modality of regulated cell death that is distinct from traditional cell death forms, characterized by abnormal accumulation of iron-dependent lipid peroxides, accompanied by shrunken mitochondria and reduced cristae, with morphological and biochemical features distinct from classical apoptosis, necrosis, and autophagy (6). Being the core organ governing iron and lipid metabolism, the liver provides a microenvironment in which HCC cells are often exposed to high iron load and oxidative stress, rendering them particularly susceptible to ferroptosis (7–9). Numerous studies have demonstrated that inducing ferroptosis—by inhibiting the SLC7A11/GPX4 axis, promoting oxidation of polyunsaturated fatty acids, or disturbing iron homeostasis—can significantly enhance the sensitivity of tumor cells to targeted agents such as sorafenib, providing a new avenue for overcoming drug resistance and recurrence in HCC (10, 11). However, the fine regulatory mechanisms of ferroptosis in HCC remain incompletely understood, particularly with respect to which upstream molecules participate in controlling the ferroptosis threshold through ubiquitination. Among them, E3 ubiquitin ligases, which determine the stability and degradation of key signaling proteins, are considered potential hub regulators in ferroptosis, yet relevant studies are still limited, which to some extent restricts the clinical translation of ferroptosis-related targets in HCC.

Encompassing a class of E3 ubiquitin ligases, the TRIM (tripartite motif-containing) protein family is defined by a conserved RING domain, one or two B-box zinc-finger domains, and a coiled-coil region; to date, over 70 members of this family have been identified (12, 13). By mediating substrate-specific ubiquitination, Members of the TRIM protein family participate extensively in essential biological processes, such as cell cycle modulation, DNA damage repair responses, and innate immune signaling pathways, and have been shown to play important regulatory roles in the development of various malignancies and immune-related diseases (14).

Among the many TRIM family members, TRIM25 is one of the most extensively studied. Early work defined TRIM25 as a classical antiviral factor that amplifies antiviral responses by mediating K63-linked polyubiquitination of RIG-I and activating downstream interferon signaling (15). Subsequent evidence has demonstrated that the functions of TRIM25 extend far beyond antiviral immunity: in multiple solid tumors, such as breast, prostate, bladder cancer and HCC, TRIM25 is frequently overexpressed and can promote tumor cell proliferation, migration and invasion by modulating p53, NF-κB and multiple stress response pathways, as well as mediating resistance to diverse anticancer therapeutic strategies (16). Notably, TRIM25 has recently been increasingly implicated in the regulation of ferroptosis. Emerging studies suggest that TRIM25 can fine-tune Nrf2-mediated antioxidant defense by promoting ubiquitin-dependent degradation of key redox-related proteins, thereby elevating the cellular tolerance threshold to lipid peroxidation damage (1). For example, in models of central nervous system and urinary tract tumors, the TRIM25–Keap1–Nrf2 axis has been shown to attenuate ferroptosis-related lipid peroxidation, reduce the cytotoxicity of ferroptosis inducers, and correlate closely with chemoresistant phenotypes. These findings collectively indicate that TRIM25 may act as a critical upstream node linking ubiquitination, oxidative stress responses, and ferroptosis sensitivity, serving as a “rheostat” in the decision between survival and death of tumor cells (17).

In hepatocellular carcinoma, TRIM25 has been shown to promote Keap1 degradation and thereby constitutively activate Nrf2 signaling, enhancing the antioxidant capacity of tumor cells and facilitating tumor progression. However, current studies have mainly focused on the regulation of the Keap1–Nrf2 axis and oxidative stress by TRIM25 (18). A comprehensive evaluation of its global expression profile in HCC tissues along with its systematic correlation to clinical disease stage as well as prognosis is still lacking; in particular, whether TRIM25 directly influences the ferroptosis sensitivity of HCC cells by reshaping iron homeostasis and lipid peroxidation remains to be clearly elucidated (13).

Taken together, TRIM25 is likely to be a key regulatory factor that links ubiquitination, oxidative stress responses, and ferroptosis sensitivity, yet its expression pattern, prognostic significance, and functional role in HCC require systematic clarification. The present study focuses on the expression pattern of TRIM25 in HCC, its association with clinical outcomes, and its impact on ferroptosis-related phenotypes, with the aim of uncovering its potential oncogenic role and molecular mechanisms and providing a candidate target for prognostic stratification and ferroptosis-based therapeutic strategies for HCC.

## 2 Materials and methods

### 2.1 Bioinformatics analysis

RNA-seq data for hepatocellular carcinoma and corresponding clinical data were downloaded from The Cancer Genome Atlas (TCGA, https://portal.gdc.cancer.gov/) database, including transcriptome data from 371 HCC tumor tissue samples and 50 corresponding adjacent normal tissue samples. In addition, HCC-related core transcriptome datasets were obtained from the International Cancer Genome Consortium (ICGC, https://dcc.icgc.org/). Data preprocessing was performed using R software: the edgeR package was used to normalize RNA-seq data and remove low-abundance genes, while the limma package was employed to clean clinical data and exclude cases with missing key information such as TNM stage or OS. Ultimately, 331 TCGA cases (including 50 adjacent tissues) and 442 ICGC cases were retained for further analyses.

Based on the ICGC cohort, TRIM25 expression levels were contrasted between 240 HCC tumor specimens and 202 matched adjacent normal tissues via the Wilcoxon rank-sum test, and boxplots were constructed using the ggplot2 package. In the TCGA cohort, variations in TRIM25 expression across patients with distinct TNM stages (stage I, n = 181; stage II, n = 92; stage III, n = 45; stage IV, n = 13) were evaluated using the Kruskal–Wallis H test, and corresponding boxplots were plotted. Furthermore, TCGA-HCC patients were divided into high- and low-TRIM25 expression subgroups according to the median expression value. Kaplan–Meier survival curves for OS were generated, and differences between groups were assessed using the log-rank test. A univariable Cox proportional hazards regression model was then constructed to evaluate the association between TRIM25 expression and OS, calculating the HR and 95% CI. Survival analyses were conducted with the survival package, and survival curves were visualized using the survminer package. *P* < 0.05 in two-sided tests was deemed statistically significant.

### 2.2 Cell lines and culture conditions

HepG2 and Huh-7 cells were retrieved from −80 °C storage and rapidly thawed in a 37 °C water bath. Cells were then transferred into complete DMEM (KeyGEN, KGL1206-500) containing 10% FBS (Vazyme, China) and centrifuged at 1,000 rpm for 5 min. After discarding the supernatant, the cell pellet was resuspended in fresh DMEM supplemented with 10% FBS and 1% penicillin–streptomycin, and seeded into culture flasks. Cells were maintained at 37 °C in a 5% CO_2_ incubator and subcultured upon reaching 70%–80% confluence.

### 2.3 Human tissue samples

Tumor tissues and matched adjacent non-tumor liver tissues (≥5 cm from the tumor margin, pathologically confirmed free of tumor cell infiltration) were collected from 12 patients who underwent radical hepatectomy at Shuguang Hospital Affiliated to Shanghai University of Traditional Chinese Medicine in 2023- 2025. All patients were diagnosed with primary hepatocellular carcinoma according to the 2022 WHO Classification of Tumors of the Digestive System and had not received any prior antitumor therapy. This study was approved by the Ethics Committee of Shuguang Hospital Affiliated to Shanghai University of Traditional Chinese Medicine (Approval No. 2023-1387-154-01), and written informed consent was obtained from all patients. Fresh tissue samples were rinsed with pre-chilled PBS. A portion of each specimen was fixed in 4% paraformaldehyde for immunohistochemistry, while the remaining tissue was immediately snap-frozen and stored at −80 °C for subsequent protein and nucleic acid extraction.

### 2.4 Western blot analysis

Approximately 50 mg of frozen tissue was homogenized in RIPA lysis buffer containing 1% PMSF using a homogenizer (60 Hz, 60 s each, three cycles) and lysed on ice for 15 min. The lysates were centrifuged at 12,000 rpm for 15 min at 4 °C, and the supernatants were collected. Protein concentrations were determined using a BCA protein assay kit. After adding 5× loading buffer, samples were adjusted to equal protein concentrations and boiled at 100 °C for 5 min for denaturation.

Following protein quantification, proteins were separated on a 10% SDS-PAGE gel (Epizyme, PG222, China) and transferred to a PVDF membrane using methanol. Membranes were blocked with rapid blocking buffer (Epizyme, PS108P) at room temperature for 10 min and then incubated overnight at 4 °C with primary antibodies against TRIM25 (Abclonal, A4347; 1:1,000) and GAPDH (Abclonal, A19056; 1:5,000). After three washes with TBST (10 min each), membranes were incubated with HRP-conjugated secondary antibody (Abclonal, AS014; 1:5,000) at room temperature for 1 h. Protein bands were visualized using an ECL chemiluminescence kit and imaged with a chemiluminescence detection system. Band densities were quantified via ImageJ software, and the relative protein expression level of TRIM25 was calculated as the ratio between the band intensity of TRIM25 and that of GAPDH.

### 2.5 Real-time quantitative PCR

Total RNA was extracted from approximately 30 mg of frozen tissue using a commercial kit following the manufacturer’s protocol. RNA concentration and purity were assessed with a microplate reader; samples with an A_260_/A_2>80_ ratio of 2.0 ± 0.2 were used for subsequent analysis. Reverse transcription was performed using a thermal cycler under the following conditions: 37 °C for 15 min, followed by 85 °C for 5 min. The resulting cDNA was diluted 10-fold with double-distilled water and stored at −20 °C.

Primers were designed using the Primer3Plus website and verified by NCBI BLAST. The sequences were as follows: TRIM25 forward: 5′-cacaagaacacggtgctgtg-3′, reverse: 5′-tctctgaggcgtccaagaga-3′; GAPDH forward: 5′-tccaaaatcaagtggggcga-3′, reverse: 5′-ctcagtgtagcccaggatgc-3′. Quantitative real-time PCR was performed using a 20 μL reaction system prepared with a SYBR Green kit on a real-time PCR system. GAPDH was used as the endogenous control, and relative TRIM25 mRNA expression was calculated using the 2^−ΔΔCt method.

### 2.6 Immunohistochemistry

Paraffin-embedded tissue sections were placed on a slide warmer at 70 °C for 20 minutes. After pre-warming, sections were dewaxed in xylene twice for 10 minutes each, then rehydrated through a graded ethanol series (100%, 95%, 80%, and 70%), and rinsed with distilled water. Endogenous peroxidase activity was blocked with 3% hydrogen peroxide for 10 minutes, followed by three 5-minute washes with PBS. Antigen retrieval was performed using citrate buffer under microwave irradiation for 10 minutes. After cooling to room temperature, tissue regions were circled with an immunohistochemistry pen and blocked with 5% bovine serum albumin at 37 °C for 30 minutes.

Sections were incubated overnight at 4 °C with anti-TRIM25 primary antibody in a humidified chamber. After three washes, they were incubated with HRP-labeled secondary antibody for 30 minutes, then washed again. Color was developed using DAB chromogen solution at room temperature, and the reaction was monitored under a light microscope and terminated with PBS. Sections were counterstained with hematoxylin for 30 seconds, rinsed with running water, differentiated, and blued with ammonia solution for 3–5 minutes. After rinsing, sections were dehydrated through graded ethanol, cleared in xylene, and mounted with neutral resin.

Images were acquired using a whole-slide scanner. The integrated optical density of TRIM25-positive areas was quantified using ImageJ software, and net values after background subtraction were used to reflect relative TRIM25 expression levels.

### 2.7 Construction of TRIM25 knockdown and overexpression cell models

To assess the effect of TRIM25 expression on the biological behaviors of HCC cells, transient transfection models of TRIM25 overexpression and silencing were established in HepG2 and Huh-7 cells using TRIM25 overexpression plasmids and short hairpin RNAs (shRNAs). Cells were divided into the following groups: vector control (VC), TRIM25 overexpression (TRIM25-OE), and TRIM25 knockdown (TRIM25-sh).

Logarithmic-phase cells were seeded into 6-well culture plates. When the cell confluence attained 60%–70%, transfection complexes were prepared in serum-free DMEM by mixing 4 μg plasmid DNA with 8 μL Lipofectamine 3000 (Invitrogen) per well and incubating at room temperature for 15 min. The original medium was then replaced with the transfection mixture and cells were incubated for 6 h, followed by replacement with complete medium supplemented with 10% FBS. Cells were collected 24–72 hours post-transfection for subsequent experimental procedures, and TRIM25 knockdown or overexpression efficiency was verified by WB and RT-qPCR.

### 2.8 Colony formation assay

To evaluate whether TRIM25 expression affects the proliferative capacity of liver cancer cells, transfected cells were seeded at an appropriate density in six-well plates and cultured at 37 °C in a 5% CO_2_ incubator for approximately 10–14 days until visible colonies formed, with each colony containing at least 50 cells in the control group. The medium was discarded, and the cells were washed with PBS, fixed with 4% paraformaldehyde for 15 min, and stained with 0.3% crystal violet solution for 30 min. After staining, residual dye was washed off with PBS. Following air-drying, images were captured, and colonies were quantified using ImageJ software.

### 2.9 Cell viability assay (CCK-8)

Prepare the CCK-8 working solution according to the instructions to achieve a final concentration of 10%. Discard the original medium, add 100 μL of CCK-8 working solution, and incubate at 37 °C in the dark for 1–2 hours. Finally, measure the optical density (OD_450_) of each well at 450 nm using a microplate reader. Calculate the change in cell viability for each group relative to the control group and blank wells.

### 2.10 Measurement of glutathione (GSH) content

Intracellular GSH content was measured using a GSH assay kit (Beyotime, S0053). Seventy-two hours after transfection, cells from each group were harvested by trypsinization (Gibco, 25200-072) and collected by centrifugation at 1,000 rpm for 5 min. Three volumes of protein-removal reagent were added to the cell pellets based on pellet volume, followed by two rapid freeze–thaw cycles between liquid nitrogen and a 37 °C water bath. Samples were then kept on ice for 5 min and centrifuged at 10,000 rpm for 5 min, and the supernatants were collected for analysis. Working solutions and standard solutions were assembled following the manufacturer’s protocols. After incubating at room temperature in the dark for 5 minutes, measure the absorbance of each group at 412 nm wavelength and plot a standard curve. Calculate GSH concentration based on the standard curve and the instructions. Simultaneously perform protein concentration quantification. The final result measures GSH concentration as the GSH content per unit of protein.

### 2.11 Measurement of malondialdehyde content

Intracellular MDA levels were measured using an MDA assay kit. Seventy-two hours after transfection, cells were harvested and lysed in RIPA lysis buffer on ice for 15 min. The lysates were centrifuged at 12,000 rpm for 10 min at 4 °C, and the supernatants were collected for BCA protein quantification and subsequent assays. Samples, standards, and blanks were prepared and mixed with working solution according to the manufacturer’s instructions and heated at 100 °C for 15 min. After cooling to room temperature, samples were centrifuged at 1,000 rpm for 10 min. The supernatant was transferred into a 96-well plate, and absorbance at 532 nm was measured immediately. MDA concentrations were calculated from a standard curve and normalized to protein content, and results are expressed as nmol/mg protein.

### 2.12 Measurement of superoxide dismutase activity

SOD activity was assessed using a commercial SOD activity assay kit according to the manufacturer’s instructions. Seventy-two hours after transfection, cells were washed twice with PBS, lysed on ice for 10–15 min, and centrifuged at 12,000 rpm for 10 min at 4 °C. The supernatants were collected for analysis. Working solutions and standards were prepared following the kit protocol. Absorbance was measured at the specified wavelength, and SOD activity was calculated using the formula provided by the manufacturer and expressed as U/mg protein.

### 2.13 Measurement of intracellular ferrous iron using FerroOrange

Intracellular Fe²^+^ levels were measured using the fluorescent probe FerroOrange. Twenty-four hours after transfection, cells from each group were washed twice with PBS and resuspended in serum-free DMEM. FerroOrange working solution (1 μM) was prepared in culture medium according to the manufacturer’s protocol. After removing the original medium, cells were incubated with the probe-containing working solution at 37 °C in a 5% CO_2_ incubator for 30 min in the dark. At the end of incubation, cells were gently washed 2–3 times with PBS and maintained in an appropriate volume of serum-free medium to keep them moist. Fluorescence images were acquired using a fluorescence inverted microscope (excitation 543–561 nm, emission 570–590 nm). ImageJ software was utilized to quantify the mean fluorescence intensity of each visual field, and the vector group was set as the control to compare the relative Fe²^+^ levels across different treatment groups.

### 2.14 Measurement of intracellular ROS levels

Intracellular ROS levels were assessed using the fluorescent probe DCFH-DA. Twenty-four hours after transfection, cells were washed twice with PBS. DCFH-DA was diluted in serum-free DMEM at 1:1,000 to a final concentration of 10 μM, and cells were incubated with the probe at 37 °C for 20 min in the dark. Excess probe was removed by washing with PBS. Images were captured under an inverted fluorescence microscope with excitation at 488 nm and emission at 525 nm, with at least three random fields collected per group. Fluorescence intensity was quantified using ImageJ software and compared with the vector control group.

### 2.15 Flow cytometric analysis of lipid peroxidation (Liperfluo)

Flow cytometry was used to detect lipid peroxidation levels using the Liperfluo probe. At 24 hours post-transfection, cells were washed twice with PBS, trypsinized, and collected by centrifugation. The Liperfluo working solution was prepared, and cells were resuspended in it and incubated at 37 °C in the dark for 30 minutes. After incubation, cells were centrifuged, the supernatant discarded, and washed three times with PBS. The cells were then resuspended in PBS, and probe fluorescence intensity was measured by flow cytometry with excitation at 490 nm and emission at 530 nm. A total of 10,000 events were recorded per sample. Mean fluorescence intensity was analyzed using FlowJo software. The Vector group was used as the baseline for comparing lipid peroxidation levels across groups.

### 2.16 Statistical analysis

Statistical analyses were performed using SPSS Statistics 27.0 from IBM in Armonk, NY, USA. Quantitative data are presented as mean plus or minus standard deviation. Normality and homogeneity of variance were assessed prior to hypothesis testing. For comparisons between two groups, an independent two-tailed Student’s t-test was applied when data satisfied the assumptions of normal distribution and equal variances; otherwise, an appropriate non-parametric test was used. For comparisons among multiple groups, one-way analysis of variance was performed when parametric assumptions were met, followed by suitable *post hoc* multiple-comparison tests. Unless otherwise specified, all *in vitro* experiments were independently repeated at least three times. A two-sided P value less than 0.05 was considered statistically significant.

## 3 Results

### 3.1 TRIM25 is highly expressed in HCC and associated with poor prognosis

**Figure 1:**
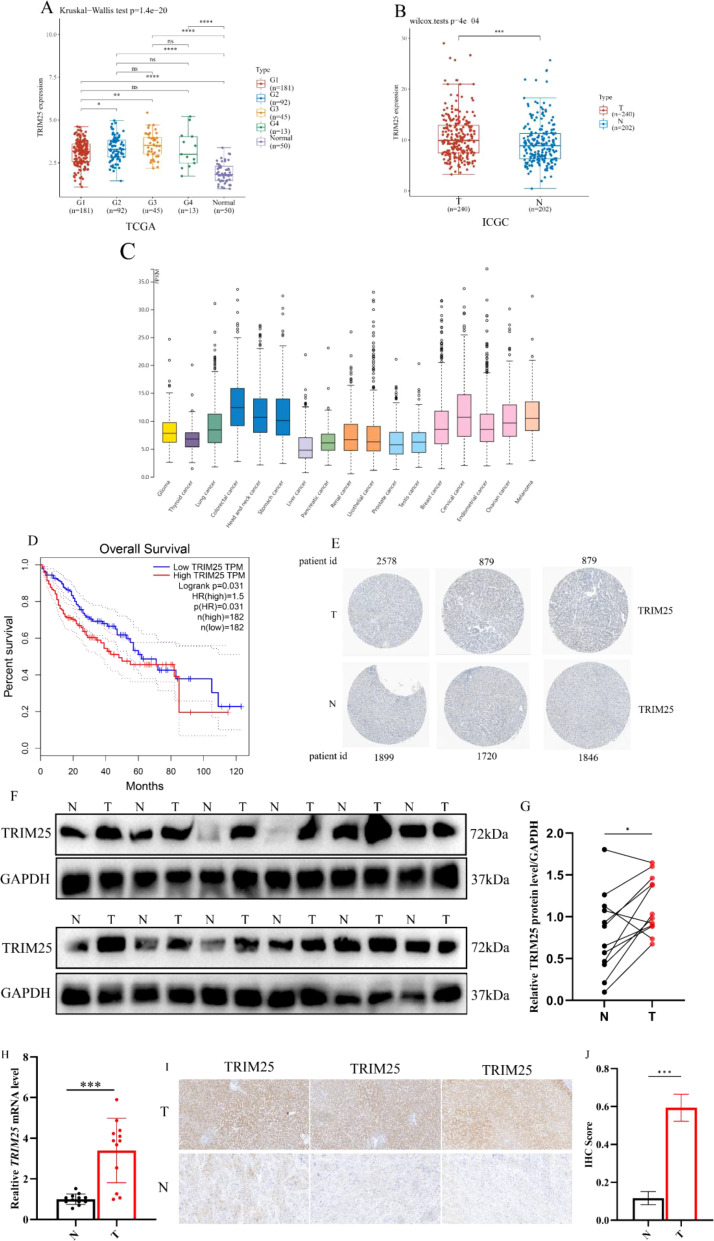
TRIM25 expression profile and its clinical relevance in HCC. **(A)** TRIM25 mRNA expression in HCC tumor tissues and adjacent normal liver tissues, and its distribution across different TNM stages in the TCGA-LIHC cohort. **(B)** TRIM25 mRNA expression in HCC tumor tissues and adjacent normal tissues in the ICGC cohort. **(C)** TRIM25 mRNA expression profile based on TCGA pan-cancer analysis. **(D)** Kaplan–Meier overall survival (OS) curves and log-rank test for TCGA-HCC patients stratified into high- and low-expression groups according to the median TRIM25 expression level. **(E)** Representative immunohistochemistry (IHC) images of TRIM25 staining in normal liver tissue and HCC tissue from the Human Protein Atlas (HPA) database. **(F)** Representative Western blot bands of TRIM25 and GAPDH in paired HCC tumor and adjacent non-tumor tissues from 12 patients in our center. **(G)** Densitometric quantification of Western blot bands showing the relative TRIM25 protein expression in paired tumor and adjacent tissues. **(H)** Relative TRIM25 mRNA expression in 12 pairs of HCC and adjacent tissues detected by RT-qPCR. **(I)** Representative IHC images of TRIM25 in HCC tissues and matched adjacent liver tissues from our center. **(J)** Semi-quantitative analysis of IHC staining presented as integrated optical density (IOD).Data are presented as mean ± standard deviation; *P < 0.05, **P < 0.01, ***P < 0.001.

To systematically characterize the expression profile of TRIM25 in hepatocellular carcinoma (HCC) and its correlation with the prognostic outcomes of patients, we first analyzed the TCGA-LIHC cohort. Compared with adjacent normal liver tissues, TRIM25 mRNA levels were significantly elevated in HCC tumor tissues and showed a gradual increase with advancing TNM stage (**Figure 1A**). Survival analysis indicated that, when patients were stratified into high- and low-expression groups according to the median TRIM25 expression level, those in the high-expression group had a markedly shorter overall survival (OS), with the difference between them showing statistical significance (**Figure 1D**).

To verify the stability and robustness of these findings, an external validation was performed using the ICGC cohort. Consistently, TRIM25 expression was also significantly higher in tumor tissues than in adjacent normal tissues in the ICGC dataset (**Figure 1B**). On this basis, we further examined surgical specimens from 12 HCC patients collected at our center. Western blot analysis showed that TRIM25 protein levels were markedly higher in tumor tissues than in matched adjacent tissues (**Figures 1F, G**), and RT-qPCR results likewise demonstrated significantly increased TRIM25 mRNA expression in tumor tissues (**Figure 1H**). Immunohistochemistry further confirmed that TRIM25 was diffusely or strongly expressed in HCC tissues but weakly expressed in adjacent non-tumor liver tissues, and semi-quantitative analysis was consistent with the Western blot data (**Figures 1I, J**). Taken together, these results indicate that TRIM25 is persistently overexpressed in HCC and is closely associated with poor patient prognosis.

### 3.2 TRIM25 knockdown inhibits HCC cell proliferation and enhances ferroptosis-related phenotypes

**Figure 2:**
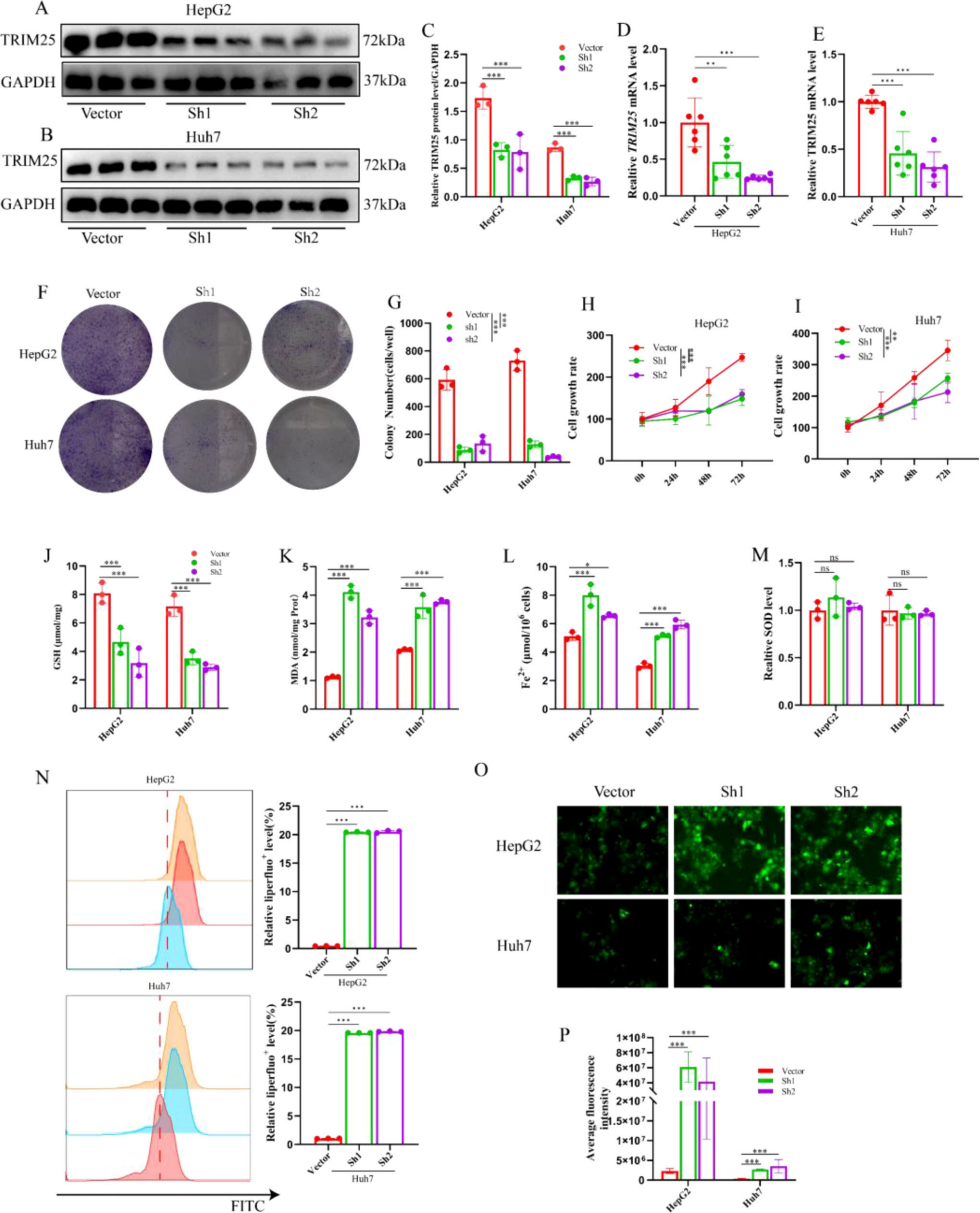
Effects of TRIM25 knockdown on HCC cell proliferation and ferroptosis-related phenotypes. **(A–C)** Western blot analysis of TRIM25 knockdown efficiency in HepG2 and Huh-7 cells: representative bands for TRIM25 and GAPDH **(A, B)** and densitometric quantification of TRIM25 relative protein levels. **(D, E)** RT-qPCR analysis of relative TRIM25 mRNA expression in HepG2 **(D)** and Huh-7 **(E)** cells. **(F)** Representative images of colony formation assays for HepG2 and Huh-7 cells under Vector, Sh1 and Sh2 conditions. **(G)** Quantitative analysis of colony numbers in each group. **(H, I)** CCK-8 cell proliferation curves of HepG2 **(H)** and Huh-7 **(I)** cells at the indicated time points. **(J)** Quantitative analysis of intracellular reduced glutathione (GSH) levels in HepG2 and Huh-7 cells. **(K)** Quantitative analysis of malondialdehyde (MDA) levels. **(L)** Quantitative analysis of intracellular ferrous iron (Fe²^+^) levels. **(M)** Quantitative analysis of superoxide dismutase (SOD) activity. **(N)** Flow cytometric histograms of lipid peroxidation detected by the Liperfluo probe in HepG2 and Huh-7 cells, and quantitative analysis of the proportion of Liperfluo^+^ cells. **(O)** Representative fluorescence images of intracellular reactive oxygen species (ROS) in HepG2 and Huh-7 cells labeled with DCFH-DA (Vector, Sh1 and Sh2 group. **(P)** Quantitative analysis of mean fluorescence intensity of ROS based on DCFH-DA staining. Data are presented as mean ± standard deviation; *P < 0.05, **P < 0.01, ***P < 0.001.

To further explore the relationship between TRIM25 and ferroptosis, TRIM25 knockdown models were established in HepG2 and Huh-7 cells, with an empty vector group (Vector) as control. Western blot and RT-qPCR analyses confirmed that TRIM25 protein and mRNA levels were significantly reduced in the sh-TRIM25 group compared with the control group, indicating successful construction of the knockdown models (**Figures 2A–E**).

Functional assays results indicated that, in both the CCK-8 and colony formation assays, the proliferative activity of sh-TRIM25 cells was significantly decreased, and the number of colonies was markedly reduced compared with the Vector group (**Figures 2F–I**), suggesting that TRIM25 knockdown suppresses HCC cell growth. To determine whether this inhibitory effect is related to ferroptosis, multiple ferroptosis-related biochemical parameters were further assessed. Compared with the Vector group, intracellular ferrous iron (Fe²^+^) levels were notably elevated in the sh-TRIM25 group (**Figure 2L**), fulfilling the key “iron dependence” feature of ferroptosis. Levels of malondialdehyde (MDA), a terminal product of lipid peroxidation, were also markedly elevated (**Figure 2K**), indicating exacerbated lipid peroxidation damage. Meanwhile, intracellular levels of the major antioxidant glutathione (GSH) and the activity of superoxide dismutase (SOD) were both significantly decreased (**Figures 2J, M**), suggesting impairment of the cellular antioxidant defense system.

Liperfluo staining revealed enhanced lipid peroxidation signals in sh-TRIM25 cells (**Figure 2N**). Consistent with the above findings, DCFH-DA fluorescence assays showed that a significant increase in total intracellular ROS levels was observed in the sh-TRIM25 group compared to the Vector group (**Figure 2O**), indicating that TRIM25 knockdown leads to substantial ROS accumulation, providing abundant oxidative substrates for lipid peroxidation reactions.

Taken together, HCC cells with TRIM25 knockdown exhibited typical ferroptosis-related phenotypes, including accumulation of ferrous iron, exacerbated lipid peroxidation, and compromised antioxidant defenses, accompanied by significantly suppressed cell proliferation. These findings collectively suggest that TRIM25 knockdown markedly enhances the ferroptosis sensitivity of HCC cells.

### 3.3 TRIM25 overexpression promotes HCC cell proliferation and attenuates ferroptosis-related phenotypes

**Figure 3:**
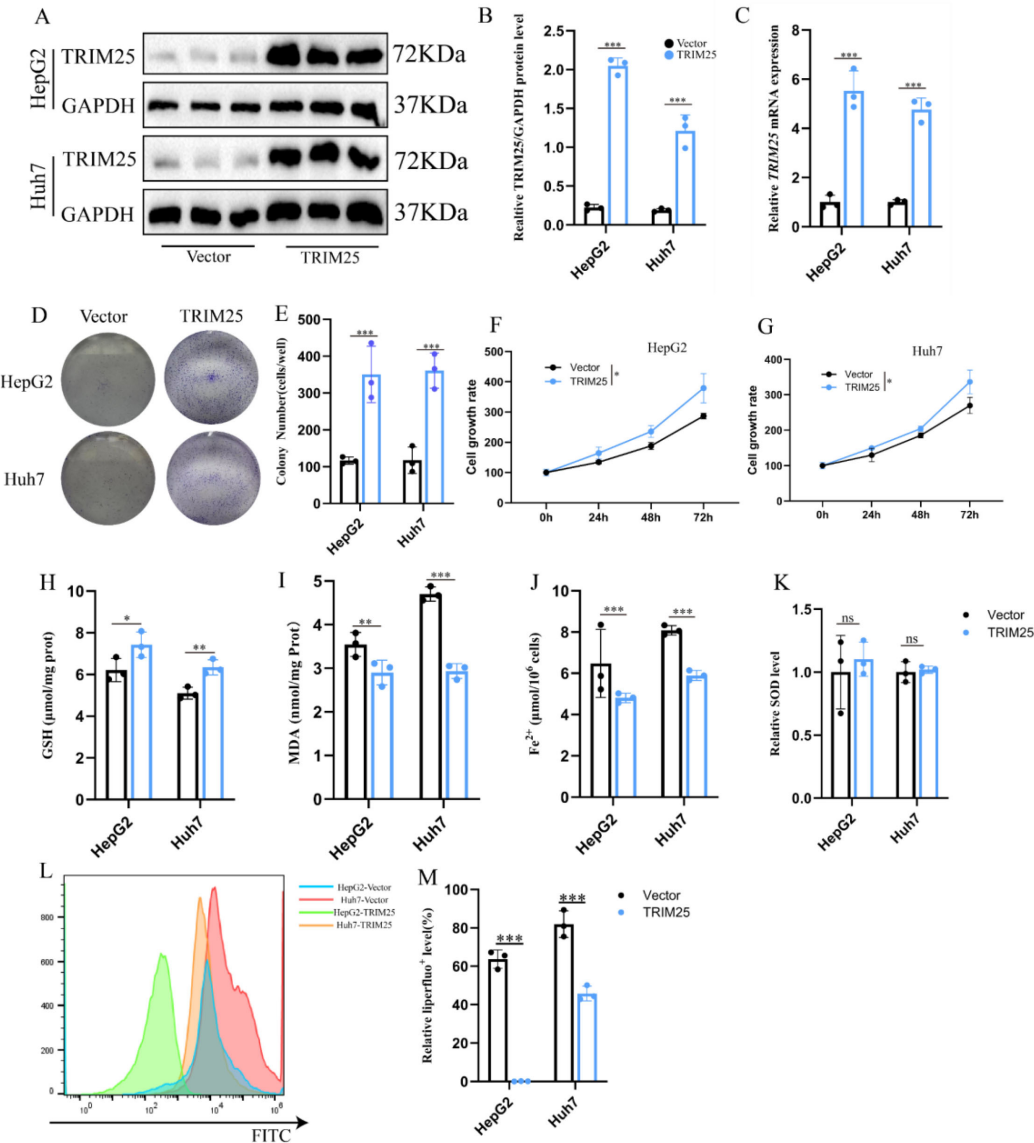
TRIM25 overexpression promotes hepatocellular carcinoma cell proliferation and attenuates ferroptosis-related phenotypes. **(A, B)** Western blot analysis of TRIM25 overexpression efficiency in HepG2 and Huh-7 cells, with representative bands and densitometric quantification. **(C)** RT-qPCR analysis of relative TRIM25 mRNA expression in HepG2 and Huh-7 cells after TRIM25 overexpression. **(D)** Representative images of colony formation assays for HepG2 and Huh-7 cells under Vector and TRIM25-OE conditions. **(E)** Quantitative analysis of colony formation showing the number of colonies in each group. **(F, G)** CCK-8 proliferation curves of HepG2 **(F)** and Huh-7 **(G)** cells at the indicated time points. **(H)** Quantitative analysis of intracellular reduced glutathione (GSH) levels in HepG2 and Huh-7 cells. **(I)** Quantitative analysis of malondialdehyde (MDA) levels, reflecting lipid peroxidation status. **(J)** Quantitative analysis of intracellular ferrous iron (Fe²^+^) levels. **(K)** Quantitative analysis of superoxide dismutase (SOD) activity. **(L)** Flow-cytometric histograms of lipid peroxidation detected by the Liperfluo probe in HepG2 and Huh-7 cells, and quantification of the proportion of Liperfluo^+^ cells.**(M)** Quantitative analysis of mean fluorescence intensity (MFI) based on Liperfluo signals. Data are presented as mean ± standard deviation; *P < 0.05, **P < 0.01, ***P < 0.001.

To further validate the regulatory effect of TRIM25 on ferroptosis sensitivity in HCC cells, TRIM25 overexpression models were constructed in HepG2 and Huh-7 cells. Western blot and RT-qPCR analyses demonstrated that TRIM25 protein and mRNA levels were significantly increased in the TRIM25 overexpression group compared with the Vector group, confirming successful model establishment (**Figures 3A–C**).

Results of the CCK-8 and colony formation assays showed that TRIM25 overexpression enhanced the proliferative activity of HCC cells and increased their colony-forming ability compared with controls (**Figures 3D–G**), indicating that upregulation of TRIM25 favors HCC cell growth. Assessment of ferroptosis-related indices revealed that TRIM25 overexpression significantly reduced intracellular ferrous iron and MDA levels (**Figures 3I, J**), suggesting decreased iron load and attenuated lipid peroxidation. In contrast, GSH levels and SOD activity were markedly increased (**Figures 3H, K**), indicating a strengthened antioxidant defense system.

Flow cytometric analysis of Liperfluo fluorescence further showed that lipid peroxidation levels were markedly lower in TRIM25-overexpressing cells than in Vector controls (**Figures 3L, M**), which was highly consistent with the observed changes in biochemical parameters.

Overall, these findings indicate that TRIM25 overexpression not only promotes HCC cell proliferation but also reduces intracellular iron load, alleviates lipid peroxidation, and enhances antioxidant defenses, thereby markedly attenuating ferroptosis-related phenotypes and ultimately decreasing the ferroptosis sensitivity of HCC cells.

## 4 Discussion

In this study, we systematically evaluated the expression pattern of TRIM25 in hepatocellular carcinoma and its impact on ferroptosis-related phenotypes using public databases, clinical specimens, and *in vitro* functional experiments. We first demonstrated that TRIM25 is consistently overexpressed in HCC tissues from both the TCGA and ICGC cohorts, with expression levels increasing in parallel with tumor stage, and that high TRIM25 expression is significantly associated with shorter overall survival (19, 20). These findings suggest that TRIM25 may be involved in the malignant progression of HCC and hold potential prognostic value. In our institutional cohort of 12 paired HCC and adjacent non-tumor tissues, TRIM25 protein and mRNA levels were also markedly elevated in tumor tissues, and IHC showed diffuse or strong positive staining in tumor cells, in line with the public database results. On this basis, functional experiments revealed that TRIM25 knockdown inhibited HCC cell proliferation and induced a series of typical ferroptosis-related biochemical changes, whereas TRIM25 overexpression exerted opposite effects. Collectively, these data indicate that high TRIM25 expression increases the tolerance threshold of HCC cells to ferroptosis, thereby promoting tumor cell survival (21).

Previous studies have shown that TRIM family members are extensively implicated in tumorigenesis, tumor progression, and cell fate decisions. They can modulate cell proliferation and apoptosis by regulating p53, NF-κB, and DNA damage response pathways, and can also promote tumor progression by shaping innate immunity and the inflammatory microenvironment (22). As one of the most extensively studied TRIM proteins, TRIM25 was initially identified as a classical antiviral factor, but has since been recognized as an oncogenic regulator in a variety of solid tumors (23). In HCC, TRIM25 has been reported to promote Keap1 degradation and constitutively activate Nrf2 signaling, thereby enhancing antioxidant capacity and accelerating tumor growth. However, these studies have mainly focused on oxidative stress and cell survival and have not systematically examined the role of TRIM25 from the perspective of ferroptosis as a novel form of regulated cell death (24). Building on previous work, the present study adds two important layers of evidence: first, by combining large-scale TCGA/ICGC cohorts with clinical tissue samples, we provide integrated evidence that TRIM25 is highly expressed in HCC and correlates with unfavorable prognosis; second, by assessing multiple indices related to iron homeostasis, lipid peroxidation, and antioxidant defense, we delineate the changes in ferroptosis sensitivity of HCC cells under TRIM25 modulation, thereby linking the pro-tumorigenic role of TRIM25 more closely to the ferroptosis pathway (25).

From the perspective of tumor biology and clinical translation, ferroptosis exhibits a dual-edged nature in HCC. On the one hand, the liver is the central organ for iron and lipid metabolism, and HCC cells in a high-iron, high–oxidative stress microenvironment are intrinsically close to the ferroptosis threshold, making them particularly susceptible to ferroptosis induction (26). On the other hand, tumor cells can actively “distance” themselves from ferroptosis by upregulating multiple antioxidant pathways, thereby acquiring resistance to targeted therapies and chemotherapy. Previous studies have shown that enhancing ferroptosis can increase the sensitivity of HCC to targeted agents such as sorafenib and even reverse established drug resistance (27, 28). Our findings suggest that TRIM25, as an upstream E3 ubiquitin ligase, may play a key role in regulating this “ferroptosis threshold.” Tumor cells with high TRIM25 expression exhibit lower iron burden and lipid peroxidation, together with stronger antioxidant capacity, and are thus less vulnerable to ferroptosis inducers or oxidative insults. In contrast, inhibition of TRIM25 expression disrupts this balance and significantly enhances ferroptosis-related phenotypes. Therefore, in future therapeutic strategies, TRIM25 may serve as a potential biomarker for predicting sensitivity to ferroptosis-targeted therapies and for prognostic stratification in HCC, as well as an adjunctive target in combination with targeted agents or immunotherapy (29).

Despite these insights, we acknowledge that there are several limitations in the current study as well. First, our evidence for ferroptosis is mainly based on a composite assessment of biochemical indices—including ferrous iron, MDA, GSH, SOD, ROS, and lipid peroxidation—and we did not incorporate classical ferroptosis inhibitors such as Ferrostatin-1, Liproxstatin-1, or deferoxamine in rescue experiments, nor did we perform discriminative interventions to distinguish ferroptosis from apoptosis, necrosis, or other cell death modalities. For this reason, we have deliberately used the term “ferroptosis-related phenotypes” to describe the observed changes, and the causal role of TRIM25 in ferroptosis pathways still needs to be confirmed by inhibitor/activator-based rescue experiments. Second, this study lacks *in vivo* experiments combining TRIM25 modulation with ferroptosis inducers, and thus cannot fully capture the integrated effects of TRIM25 within the tumor microenvironment and under immune system involvement. In addition, it should be noted that HepG2 is derived from a pediatric hepatoblastoma rather than a typical adult hepatocellular carcinoma. Although HepG2 has been widely used as a hepatocyte-like HCC model in ferroptosis research, its biological characteristics are not identical to those of classical HCC. To partially address this, we also included Huh-7, a bona fide HCC cell line, in all functional assays. Nevertheless, future studies should validate the role of TRIM25 in ferroptosis sensitivity across additional HCC cell lines and primary tumor cells. In addition, the number of clinical samples was relatively small, precluding comprehensive multivariable Cox regression analyses to determine whether TRIM25 is an independent prognostic factor; larger, multi-center cohorts will be needed for validation. Finally, we did not systematically dissect the direct substrate spectrum and downstream signaling network of TRIM25. Whether TRIM25 primarily acts through the Keap1–Nrf2 axis, or also involves ferritin, ferritinophagy, and other iron-handling pathways, remains to be clarified by further molecular and *in vivo* studies.

In summary, this study demonstrates that TRIM25 is persistently overexpressed in HCC and is closely associated with tumor progression and poor prognosis. Functionally, TRIM25 upregulation helps maintain a stable balance of iron load and antioxidant status in HCC cells, attenuates ferroptosis-related phenotypes, and thereby promotes cell survival and proliferation. These findings not only provide new insights into the oncogenic role of TRIM25 but also suggest that TRIM25 may represent a critical node linking ubiquitination, oxidative stress responses, and ferroptosis sensitivity, offering a promising molecular target and theoretical basis for the development of ferroptosis-based precision therapies in HCC.

## Data Availability

The raw data supporting the conclusions of this article will be made available by the authors, without undue reservation.
